# covid19census: U.S. and Italy COVID-19 metrics and other epidemiological data

**DOI:** 10.1093/database/baab027

**Published:** 2021-05-15

**Authors:** Claudio Zanettini, Mohamed Omar, Wikum Dinalankara, Eddie Luidy Imada, Elizabeth Colantuoni, Giovanni Parmigiani, Luigi Marchionni

**Affiliations:** Department of Pathology and Laboratory Medicine, Weill Cornell Medicine, 1300 York Avenue, New York, NY 10065, USA; Department of Pathology and Laboratory Medicine, Weill Cornell Medicine, 1300 York Avenue, New York, NY 10065, USA; Department of Pathology and Laboratory Medicine, Weill Cornell Medicine, 1300 York Avenue, New York, NY 10065, USA; Department of Pathology and Laboratory Medicine, Weill Cornell Medicine, 1300 York Avenue, New York, NY 10065, USA; Department of Biostatistics, Johns Hopkins University, 615 N Wolfe St, Baltimore, MD 21205, USA; Department of Data Sciences, Dana-Farber Cancer Institute, 450 Brookline Avenue, Boston, MA 02215, USA; Department of Biostatistics, Harvard T.H. Chan School of Public Health, 677 Huntington Ave, Boston, MA 02115, USA; Department of Pathology and Laboratory Medicine, Weill Cornell Medicine, 1300 York Avenue, New York, NY 10065, USA

## Abstract

Since the beginning of the coronavirus disease-2019 (COVID-19) pandemic in 2020, there has been a tremendous accumulation of data capturing different statistics including the number of tests, confirmed cases and deaths. This data wealth offers a great opportunity for researchers to model the effect of certain variables on COVID-19 morbidity and mortality and to get a better understanding of the disease at the epidemiological level. However, in order to draw any reliable and unbiased estimate, models also need to take into account other variables and metrics available from a plurality of official and unofficial heterogenous resources. In this study, we introduce covid19census, an R package that extracts from many different repositories and combines together COVID-19 metrics and other demographic, environment- and health-related variables of the USA and Italy at the county and regional levels, respectively. The package is equipped with a number of user-friendly functions that dynamically extract the data over different timepoints and contains a detailed description of the included variables. To demonstrate the utility of this tool, we used it to extract and combine different county-level data from the USA, which we subsequently used to model the effect of diabetes on COVID-19 mortality at the county level, taking into account other variables that may influence such effects. In conclusion, it was observed that the ‘covid19census’ package allows to easily extract area-level data from both the USA and Italy using few functions. These comprehensive data can be used to provide reliable estimates of the effect of certain variables on COVID-19 outcomes.

**Database URL:** https://github.com/c1au6i0/covid19census

## Introduction

In the midst of the coronavirus disease-2019 (COVID-19) pandemic, unraveling the constant flow of epidemiological data is of paramount importance, not only to guide the evaluation and implementation of non-pharmacological interventions (NPIs) but also to optimize drug development.

For this reason, during the pandemic, various private as well as government institutions have been reporting tallies of COVID-19 cases, deaths and hospitalizations aggregated at the country, state or regional level (1–6). The Johns Hopkins University was among the first to combine COVID-19 metrics obtained from different sources and countries in a centralized repository updated daily (3). The expansion of repositories and databases also led to the development of Application Programming Interfaces (APIs) that allow COVID-19 data to be easily retrieved and analyzed (5, 7–9).

These public databases and APIs had a tremendous impact on COVID-19 epidemiological research. For example, in the early phases of the pandemic, analysis and modeling of COVID-19 confirmed cases and deaths have been employed to assess the effects of NPIs in China and Europe (10, 11). More recently, the increased flow of COVID-19 data and the integration of different sources of information (seasonality of other coronaviruses and U.S. clinical care) have allowed even more long-term predictions of the feasibility and effectiveness of possible containment strategies (12). Similarly, early evidence of the correlation between Bacille Calmette–Guerin vaccination and COVID-19 outcomes spur several clinical investigations (13, 14). However, the implications and conclusions of that initial observation were curtailed by subsequent models that included more factors (15). Overall, these few examples underscore the importance of providing public access to ongoing COVID-19 metrics, which can be used in multivariable analyses to draw reliable estimates.

To this end, we developed ‘covid19census’, an R package that not only allows retrieving COVID-19 metrics (aggregated at the county/regional level) from different repositories and databases but also presents the novel feature of combining them with demographic, health- and environment-related information. By providing tools to rapidly access and combine heterogenous datasets, the package aims at facilitating multivariable analysis and modeling of COVID-19 data by the scientific community. Specific effort was made to provide a detailed documentation for each of the variables returned by the functions and to list external sources and methodology of their collection with the objective of promoting appropriate analyses.

At the current stage, the package allows to retrieve data of the USA and Italy (at the county and regional levels) since both countries had a dramatic spread of severe acute respiratory syndrome coronavirus 2 infection and were among the first to make county- and regional-level COVID-19 metrics available. However, the package has the potential to be expanded in future versions with aggregated or individual-level data from other countries.

### Algorithm and sources

**Figure 1.**
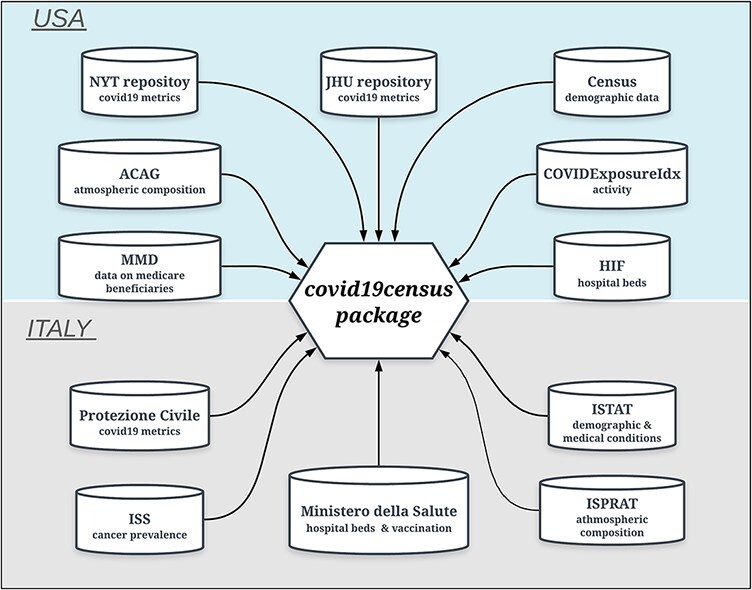
Data sources of the ‘covid19census’ package. Top: repositories for U.S.-related county data. Bottom: sources for Italian data aggregated at the regional level. Note that COVID-19 U.S. and Italian metrics are retrieved dynamically from repositories that are updated daily.

A family of ‘get’ functions is employed by the R package to dynamically extract up-to-date time-series data from different online sources and combine them to return a data frame of integrated data (Figure 1).

For the USA, the prefix of the functions to extract the data is ‘getus’, and it is followed by the specific metric of interest:

- ‘getus_covid’: extracts data of COVID-19 from the New York Times GitHub (using argument repo = ‘nyt’) repository or from the John Hopkins University GitHub repository (using argument repo = ‘jhu’) (https://github.com/CSSEGISandData).
- ‘getus_dex’: extracts data of DEX, an activity index calculated by Victor Couture and collaborators using smartphone movement data provided by PlaceIQ (https://github.com/COVIDExposureIndices/COVIDExposureIndices).
- ‘getus_tests’: extract info regarding number of performed COVID-19 tests and their results and hospitalization from the repository of the COVID Tracking Project (at the state level) (https://covidtracking.com/api%7D).
- ‘getus_all’: executes all the above functions and joins the results with other datasets statically contained in the package and returns a data frame with 326 variables.

Data regarding the household composition, population sex, race, age and poverty levels (2018) were retrieved from the American Community Survey (https://data.census.gov/cedsci/table?q=United%20States). Medical conditions, influenza and pneumococcal vaccination coverage, tobacco use, cancer and data relative to the number of medical and emergency visits (2018) of Medicare beneficiaries (i.e. people of age 65 years or older) were obtained from the Mapping Medicare Disparities tool (https://data.cms.gov/mapping-medicare-disparities).

The number of hospital beds per county (2020) was calculated from data of the Homeland Infrastructure Foundation (https://hifld-geoplatform.opendata.arcgis.com/datasets/hospitals/data?page=18). Emissions of particulate matter 2.5 (PM2.5) (2016) were reported by the Atmospheric Composition Analysis Group (http://fizz.phys.dal.ca/∼atmos/martin/?page_id=140#V4.NA.02.MAPLE).

For Italy, the prefix of the function is ‘getit’ followed by either ‘covid’ or ‘all’.

- ‘getit_covid’: extracts data of COVID-19 cases, deaths, hospitalizations and tests from the Protezione Civile (https://github.com/pcm-dpc/COVID-19).
- ‘getit_all’: executes the above function, joins the results with other datasets statically contained in the package and returns a data frame with 64 variables.

Age and sex of the population (2019), first aid and medical guard visits (2018), smoking status (2018), prevalence of chronic conditions (2018), annual household income (2017), household crowding index (2018) and body mass index were collected from ISTAT (http://dati.istat.it/?lang=en). The prevalence of types of cancer patients (2016), influenza vaccination coverage (2019) and the number of hospital beds per 1000 people (2017) were obtained from the Ministero della Salute (http://www.dati.salute.gov.it). Data of PM2.5 (2017) were obtained from the Istituto Superiore per la Protezione Ambientale (https://annuario.isprambiente.it/pon/basic/14).

The package documentation reports and describes each variable (column names) and lists all relative data sources. Because of the large number of variables and in order to facilitate exploration of the documentation, it was deemed more practical to create separate functions with separate documentation for each country.

Static U.S. and Italy datasets can be accessed directly using ‘data()’. The country that data refer to is specified in the first two letters of the object name. For example, ‘us_dem’ contains demographic information (sex and age) of U.S. counties, whereas ‘it_dem’ of regions of Italy.

The package is currently available on GitHub (https://github.com/c1au6i0/covid19census). The following code launches the functions and assigns the returned data frames to different names:

library(covid19census)dat us<-getus_all(repo=“jhu”)##US COVID-19 data up to 2021-01-03 successfully retrieved from JHU repository!##US mobility data up to 2020-12-24 successfully retrieved!##US test data up to 2021-01-03 successfully retrieved

dat it *<*-getit_all()##Italy COVID-19 data up to 2021-01-04 17:00:00successfully retrieved!

unlist(lapply(list(dat_it,datus),class))  [1]“data.frame”“data.frame”

Information of the data frames generated by the two functions are reported in Table 1

**Table 1.** Information regarding the data frames returned by the functions

	getus_all	getit_all
Number of columns/variables	324	64
Number of counties/regions	3244	21
Number of sources	7	4
Date of the first reported COVID-19 case	21 January 2020	24 February 2020

For each function, Table 1 shows the number of columns or variables; the number of unique regions (Italy) and counties (USA); the number of unique data sources and the earliest date of COVID-19 metric. Note that some of the U.S. variables are at the state level (e.g. tests).

### Example of use

Data exploration and modeling can be conveniently performed on a single data frame that contains COVID-19 data together with many other metrics retrieved from multiple sources. Here, we provide an example of how the data retrieved by ‘covid19census’ can be used to estimate the effect of certain variables on the COVID-19 outcome using robust statistical methods. Since type 1 and type 2 diabetes have been reported as significant risk factors for COVID-19-related mortality (16, 17), the package was employed to evaluate the association between diabetes prevalence and COVID-19 mortality rate, at the U.S. county level, controlling for demographic and health-related potential confounders.

Updated data of COVID-19 deaths, cases and tests as well as other demographic indices were retrieved using the function ‘getus_all’. A total of 102 variables thought to be associated with the exposure (diabetes prevalence) and outcome (COVID-19 mortality rate) or the outcome alone were selected to be included in the model as potential confounders. Some of those variables were originally expressed as absolute number of individuals (in each county) and thus were divided by county population to provide normalized and comparable values. Moreover, one more variable, the number of days since the first reported case, was calculated.

A generalized propensity score (PS) approach was employed for assessing the potential association between county-level diabetes prevalence in people older than 65 years and COVID-19 mortality, while controlling for the effects on mortality of other variables [the confounders; see the study by Austin (18) for a review on the topic]. In particular, in the current study, the PS represents the conditional probability of the prevalence of diabetes, given the variables selected, which can potentially affect the mortality by themselves. The PS was calculated by regressing the logit of diabetes prevalence on the potential confounders [PS = logit(diabetes prevalence) ∼ variable1 + variable2 *+…*] (19). Subsequently, the estimated PS was used to divide the dataset into five equal-sized strata. Stratification allows to evaluate the potential effect of diabetes prevalence on COVID-19 mortality in counties with a similar distribution of covariates and has been shown to reduce the model bias in observational studies [see the study by Austin (18)]. In each stratum, we fit a quasi-Poisson linear model using diabetes prevalence in the elderly as the exposure and COVID-19 mortality rate as the outcome, with the estimated PS and indicators of U.S. states as covariates. The county’s total population was used as an offset to account for the population size [estimate = mortality ∼ diabetes + PS + state + offset(population)].

To quantify the change in mortality associated with increments of 10% in diabetes prevalence, we calculated mortality risk ratios (MRRs) by dividing the coefficient of the model by 10 and exponentiating it (20). Finally, an average of the MRR weighted by the number of observations in each stratum was calculated together with the corresponding confidence intervals (CIs). The analysis was repeated using data up to different dates or end points to assess the stability of the estimated effect and its variation with the accumulation of new observations.

All scripts and codes used to pre-process and analyze the data are available at https://github.com/marchionniLab/covid19census_analysis.

**Figure 2.**
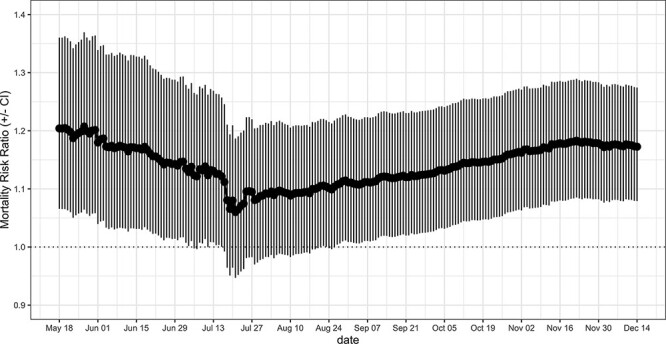
MRRs associated with an increment of 10% in county-level prevalence of diabetes and relative CIs. The weighted MRR was calculated at different end points and by including counties with at least one confirmed case.

As of 14 December 2021, after controlling for relevant demographic, health-related and environmental potential confounders, county-level prevalence of diabetes in the elderly was found to be positively associated with COVID-19 mortality. In particular, increments of 10% in diabetes prevalence were estimated to be associated with a 14.3% (95% CI: 5.2–24.1%) increase in COVID-19 mortality. The effect of diabetes prevalence on COVID-19 county mortality remained stable over the last 2 months, indicating that, at this stage, the accumulation of new data is unlikely to strongly affect the results of the model (Figure 2).

The current example illustrates the use of the package not only to investigate potential factors associated with COVID-19 metrics but also to monitor possible time or seasonal-related changes in that association.

## Conclusions

The R package ‘covid19census’ extracts and integrates epidemiological COVID-19 data from Italy and the USA at the regional and county levels, respectively, together with several other demographic and health-related indices. Currently, the data frames returned by the main functions (‘getus_all’ and ‘getit_all’) consist of 324 variables per 3244 U.S. counties and 64 variables per 21 Italian regions (19 regions and 2 autonomous provinces), respectively. By combining data from different sources, the package is aimed at promoting and simplifying the analysis and modeling of COVID-19 data by the scientific community.
